# Antitumor activity of selective MEK1/2 inhibitor AZD6244 in combination with PI3K/mTOR inhibitor BEZ235 in gefitinib-resistant NSCLC xenograft models

**DOI:** 10.1186/1756-9966-33-52

**Published:** 2014-06-17

**Authors:** Yiqing Qu, Xiuxiu Wu, Yunhong Yin, Yan Yang, Dedong Ma, Hao Li

**Affiliations:** 1 Department of Respiratory medicine, Qilu Hospital of Shandong University, 107 Wenhuaxi Road, Jinan 250012, China

**Keywords:** AZD6244, BEZ235, Tyrosine kinase inhibitor, Non-small cell lung cancer

## Abstract

**Purpose:**

Although the EGF receptor tyrosine kinase inhibitors (EGFR-TKI) gefitinib have shown dramatic effects against *EGFR* mutant lung cancer, patients become resistant by various mechanisms, including gatekeeper *EGFR*-T790M mutation, *MET* amplification, and *KRAS* mutation, thereafter relapsing. AZD6244 is a potent, selective, and orally available MEK1/2 inhibitor. In this study, we evaluated the therapeutic efficacy of AZD6244 alone or with BEZ235, an orally available potent inhibitor of phosphatidylinositol 3–kinase (PI3K) and mammalian target of rapamycin (mTOR), in gefitinib-resistant non-small cell lung carcinoma (NSCLC) models.

**Experimental design:**

NCI-H1975 with *EGFR*-T790M mutation, NCI-H1993 with *MET* amplification and NCI-H460 with *KRAS*/*PIK3CA* mutation human NSCLC cells were subcutaneous injected into the athymic nude mice respectively. Mice were randomly assigned to treatment with AZD6244, BEZ235, AZD6244 plus BEZ235, or control for 3 weeks, then all mice were sacrificed and tumor tissues were subjected to western blot analyses and immunohistochemical staining.

**Results:**

AZD6244 could inhibit the tumor growth of NCI-H1993, but slightly inhibit the tumor growth of NCI-1975 and NCI-H460. Combining AZD6244 with BEZ235 markedly enhanced their antitumor effects and without any marked adverse events. Western blot analysis and immunohistochemical staining revealed that AZD6244 alone reduced ERK1/2 phosphorylation, angiogenesis, and tumor cell proliferation. Moreover, MEK1/2 inhibition resulted in decreased AKT phosphorylation in NCI-H1993 tumor model. BEZ235 also inhibited AKT phosphorylation as well as their downstream molecules in all three tumor models. The antiangiogenic effects were substantially enhanced when the agents were combined, which may due to the reduced expression of matrix metallopeptidase-9 in tumor tissues (MMP-9).

**Conclusions:**

In this study, we evaluated therapy directed against MEK and PI3K/mTOR in distinct gefitinib-resistant NSCLC xenograft models. Combining AZD6244 with BEZ235 enhanced their antitumor and antiangiogenic effects. We concluded that the combination of a selective MEK inhibitor and a PI3K/mTOR inhibitor was effective in suppressing the growth of gefitinib-resistant tumors caused by *EGFR* T790M mutation, *MET* amplification, and *KRAS*/*PIK3CA* mutation. This new therapeutic strategy may be a practical approach in the treatment of these patients.

## Introduction

Lung cancer is the leading cause of cancer-related death in many countries, including the China [1]. Non-small cell lung cancer (NSCLC) accounts for up to 80% of all lung cancer cases; patients typically present with advanced disease at the time of diagnosis. The prognosis of patients with advanced lung cancer remains poor, and recent studies show that conventional therapies may have reached a therapeutic plateau as evidenced by the 5-year survival rate for NSCLCs, which remains at 15% [2,3]. The EGF receptor tyrosine kinase inhibitors (EGFR-TKIs) gefitinib and erlotinib have shown marked therapeutic effects against NSCLCs with *EGFR* activating mutations, such as exon 19 deletions and L858R point mutations [4]. Almost all tumors, however, acquire resistance to EGFR-TKIs after varying periods of time. Common mechanisms for acquired resistance include emergence of an *EGFR* gatekeeper mutation (T790M) and *MET* gene amplification [5,6]. In addition, *PIK3CA* mutations as well as *KRAS* mutations have been found to contribute to EGFR-TKIs resistance in a subpopulation of tumors [7,8]. The limited therapeutic options currently available for patients with advanced lung cancer create a pressing need to identify new therapeutic strategy.

Selumetinib (AZD6244) is an oral, non-ATP competitive inhibitor and highly specific for extracellular signal-regulated kinase (ERK) kinase (MEK)1/2, a key enzyme in the RAS-RAF-MEK-ERK pathway. AZD6244 had minimal effects on the p38, c-Jun-NH_2_-kinase, PI3K, and MEK5/ERK5 pathways and is currently in phase II clinical trial in *KRAS*-mutant NSCLC [9,10]. *In vivo,* AZD6244 could inhibit the tumor growth in HT-29 xenograft model, which is a colorectal tumor model carrying a *BRAF* mutation, at a dose of 100 mg/kg and the tumor growth inhibition of AZD6244 is better than gemcitabine [11]. However, the inhibition of MEK signaling alone may not be sufficient in patients with gefitinib-resistant NSCLC, and negative feedback mechanisms in PI3K pathway may be problematic when it is used alone [12]. By contrast, combined blockade of both pathways was able to overcome the reciprocal pathway activation induced by inhibitor-mediated release of negative feedback loops and resulted in a significant tumor growth inhibition. Thus, coinhibition of both pathways has shown use in reducing tumor growth in a variety of xenograft models [13,14], and clinical trials of such combinations are under way in adults.

BEZ235 is an orally available dual inhibitor of PI3K and mTOR that is being evaluated in phase I/II trials [15]. With the aim of developing effective therapeutic strategy for treatment gefitinib-resistant NSCLCs, we have initially evaluated the antitumor activity of AZD6244 alone or combination with BEZ235 in a panel of three human NSCLC cell lines, which were selected according to their different mutation status for *EGFR-*T790M*, MET* and *KRAS*/*PIK3CA* genes. We hypothesized that targeting the MEK pathway in combination with selective inhibitors of PI3K/mTOR signaling, could overcome gefitinib-resistant NSCLC and enhance the antitumor efficacy.

## Methods

### Reagents

AZD6244 and BEZ235 were purchased from Sellech Chemicals (Houston, TX, USA), all drugs were dissolved in sterile dimethylsulfoxide (DMSO) and a 10 mM working solution was prepared and stored in aliquots at -22°C. Working concentrations were diluted in culture medium just before each experiment. RPMI1640 media and fetal bovine serum (FBS) were purchased from Invitrogen (Carlsbad, CA, USA). Fibronectin and 3-(4, 5-dimethylthiazol-2-yl)-2, 5-diphenyltetrazolium bromide (MTT) were obtained from Sigma (St. Louis, MO, USA). Phospho-AKT (Ser473, p-AKT), phospho-S6 (Ser240/244, p-S6), phospho-4E-BP1 (Ser 65, p-4E-BP1), phospho-ERK1/2 (Thr202/Tyr204, p-ERK1/2), phospho-MEK1/2 (Ser217/221, p-MEK1/2), AKT, S6, 4E-BP1, MEK1/2 and ERK1/2 antibodies were purchased from Santa Cruz Biotechnology, Inc (Santa Cruz, CA, USA). CD31 and Ki-67 antibodies for IHC were purchased from Cell Signaling Technology (Danvers, MA, USA). All other chemicals used in this study were of analytical reagent grade.

### Cell lines

The NCI-H1975 *EGFR* T790M mutation [16], NCI-H460 *KRAS*/*PIK3CA* mutation and NCI-H1993 *MET* amplification [17,18] human NSCLC cell lines were obtained from American Type Culture Collection (ATCC) (Manassas, VA, USA). The cells were cultured in RPMI1640 medium supplemented with 10% FBS, 100 mg/L streptomycin, 100 IU/mL penicillin and 0.03% L-glutamine (Hyclone, Logan, UT, USA) and maintained at 37°C with 5% CO_2_ in a humidified atmosphere.

### Cell viability assay

Cell viability was measured using the MTT [3-(4,5-dimethylthiazol-2-yl)-2,5-diphenyl tetrazolium] dye reduction method. Tumor cells (1 × 10^4^ cells/100 mL/well) in RPMI1640 medium with 10% FBS were plated into 96-well plates and cultured with indicated compounds for 72 h, followed by the addition of 50 mL of MTT solution (2 mg/mL; Sigma, St. Louis, MO) to each well and further incubation for 2 h. The medium was removed, and the dark blue crystals in each well were dissolved in 100 mL dimethyl sulfoxide. The absorbance of the wells was measured with a microplate reader at test and reference wavelengths of 490 nm, respectively. Percent growth was reported relative to untreated controls. Each experiment contained at least triplicate samples and was performed at least three times.

### Efficacy study *in vivo*

BALB/C nude mice (female, 6-7 weeks old) were obtained from Vital River (Beijing, China). Mice were maintained under super pathogen-free conditions and housed in barrier facilities on a 12-h light/dark cycle, with food and water ad libitum. All animal experiments were performed in accordance with protocols approved by the Shandong University Experimental Animal Care and Use Committee. Mice were injected subcutaneous (s.c.) with 5 × 10^6^ NCI-H1993, NCI-H1975 and NCI-H460 cells that had been resuspended in 200 μL of matrigel (BD Biosciences, Milan, IT). AZD6244, solubilized in a methocel/polysorbate buffer, was injected by oral gavage twice daily at the dose of 25 mg/kg for 3 weeks [19]. BEZ235, was reconstituted in NMP (1-methyl-2 pyrrolidone) and PEG300, and injected by oral gavage once daily at the dose of 20 mg/kg for 3 weeks [20]. When the mean volumes of tumors were between 150 and 200 mm^3^, mice were randomly divided in four groups (ten mice per group). The tumor volume and body weight in each group were balanced. The animals were ear-punched for identification during the study. Two orthogonal diameters of the tumor are measured with digital vernier calipers and individual animal weights were weighed and recorded twice a week. Tumor volume (TV) are measured and recorded during treatment period by the formula: TV = Length × Width^2^/2. Growth inhibition from the start of treatment was assessed by comparison of the differences in tumor volume between control and treated groups. Tumor growth inhibition T/C ratio is calculated by the following equations: T/C ratio = (V_t_ - V_0_)_Compound treated_/(V_t_ - V_0_)_Control_ × 100 %.

### Western blot analysis

The expressions of, p-ERK1/2, p-AKT, p-S6, p-MEK1/2 and p-4E-BP1 in tumor tissues were examined by Western Blotting. Fresh tumors in each group were resected after last treatment with AZD6244 and/or BEZ235 for 2 h on Day 21 of the efficacy study. Tumor tissues were lysed by lysis buffer (50 mM HEPES [pH 7.4], 150 mM NaCl, 10% glycerol, 1% Triton X-100, 1.5 mM MgCl2, 1 mM EDTA [pH 8.0], 100 mM NaF, 1 mM phenylmethylsulfonyl fluoride 1 mM sodium orthovanadate, 10 μg/mL aprotinin, 50 μg/mL leupeptin, and 1 μg/mL pepstatin A). The resected tumor samples were homogenized with lysis buffer containing 25 mM b-glycerophosphate and 0.5% (v / v) phosphatase inhibitor cocktail 2 (Sigma-Aldrich, St. Louis, MO, USA) at 4°C. Cellular debris was removed by centrifugation at 17 860 *g* for 20 min at 4°C. Aliquots of the supernatants containing 5–20 μg of protein were subjected to SDS-PAGE under reducing conditions. The protein concentration of the supernatant was determined by Bio-Rad protein assay reagent (Bio-Rad, CA, USA). Equal amounts of protein were separated by sodium dodecyl sulfate/polyacrylamidegel electrophoresis (SDS/PAGE) on 10% gels, blotted on polyvinylidene difluoride (PVDF), and probed with p-ERK1/2, p-AKT, p-S6, p-MEK1/2, p-4E-BP1, MMP2, MMP9, ERK1/2, AKT, S6, MEK1/2 and 4E-BP1 rabbit monoclonal antibody and subsequently with goatanti-rabbit (HRP), and detected by chemiluminescence. To measure protein loading, antibodies directed against β-actin were used.

### Immunohistochemical analysis

Fresh tumors in each group were resected after last treatment with AZD6244 and/or BEZ235 for 2 h on Day 21 of the efficacy study, fixing in formalin and embedding the tumor tissue. Cutting and mounting the section. Immunocytochemical analysis was performed according to the method described on the commercial kits to examine the expressions of CD31 and Ki-67 (Cell Signaling Technology, Danvers, MA, USA).

### Caspase activity assay

The apoptotic markers, activity of caspase-3,-8 and -9 were measured by using caspase colorimetric protease kits (Abnova, Walnut, CA, USA). Fresh tumors in each group were resected after last treatment with AZD6244 and/or BEZ235 for 2 h on Day 21 of the efficacy study, and then tumor lysis containing 200 μg of protein was incubated with 5 μL of 4 mM *p*NA-conjugated substrate (DEVD-*p*NA, IETD-*p*NA and LEHD-*p*NA) at 37°C for 2 h. The amount of *p*NA released was measured at 405 nm using a microplate reader.

### Statistical analysis of the data

All results and data were confirmed in at least three separate experiments. Data are expressed as means ± SD, and were analyzed by ANOVA using Statistics Package for Social Science (SPSS) software (version 13.0; SPSS, Chicago, IL, USA). Test article can be demonstrated as an effective compound until T/C ratio ≤ 42% and a value of P < 0.05 was indicated to be statistically significance on tumor volume calculation.

## Results

### Effect of AZD6244 and BEZ235 on viability of gefitinib-resistant NSCLC *in vitro*

**Figure 1:**
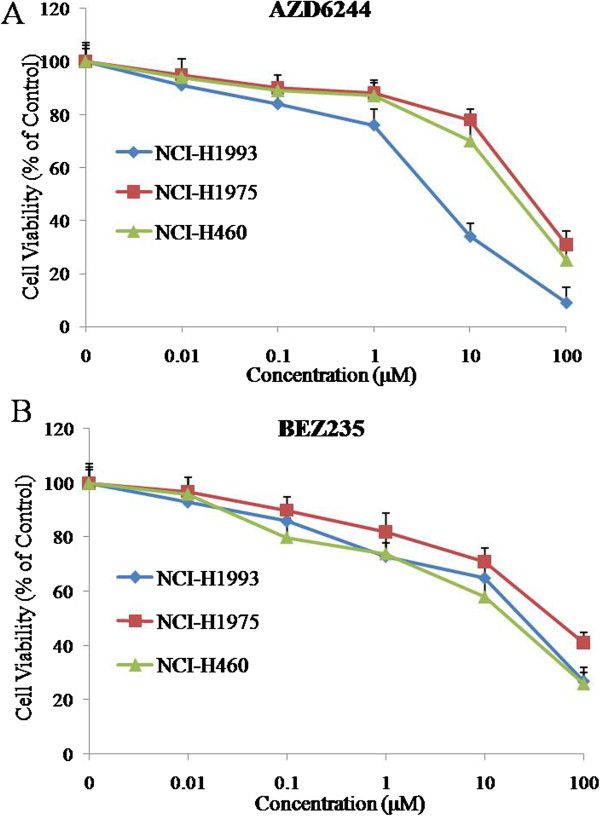
**Anti-proliferative effects of AZD6244 and BEZ235 in NCI-H1993, NCI-H1975 and NCI-H460 gefitinib-resistant cell lines.** Cells were treated with varying concentrations of AZD6244 **(A)** or BEZ235 **(B)** alone for 72 h. Doses ranged from 0.01 μM to 100 μM. Mean ± SD, n = 5.

Before evaluating the effect of AZD6244, BEZ235 and AZD6244 plus BEZ235 treatment on gefitinib-resistant NSCLC xenograft models in nude mice, the sensitivity of cell lines to compounds was evaluated *in vitro.* Cell proliferation was analyzed by MTT assay in cells treated with 0, 0.01, 0.1, 1, 10 and 100 μM of AZD6244 or BEZ235 for 72 h. The results showed that AZD6244 significantly suppressed the growth of NCI-H1993 with a low micromolar IC50 value of 5.6 μM (Figure 1A). Moreover, AZD6244 alone mildly inhibited cell growth with IC50 values of 37.5 μM and 26.8 μM in NCI-H1975 and NCI-H460 cells, respectively (Figure 1A). BEZ235 alone also suppressed the growth of three cell lines with slightly high IC50 values of 23.5, 67.8 and 16.8 μM in NCI-H1993, NCI-H1975 and NCI-H460 cells, respectively (Figure 1B).

### Concurrent inhibition of MEK and PI3K/mTOR has a synergistic effect on gefitinib-resistant NSCLC cell lines growth *in vitro*

**Figure 2:**
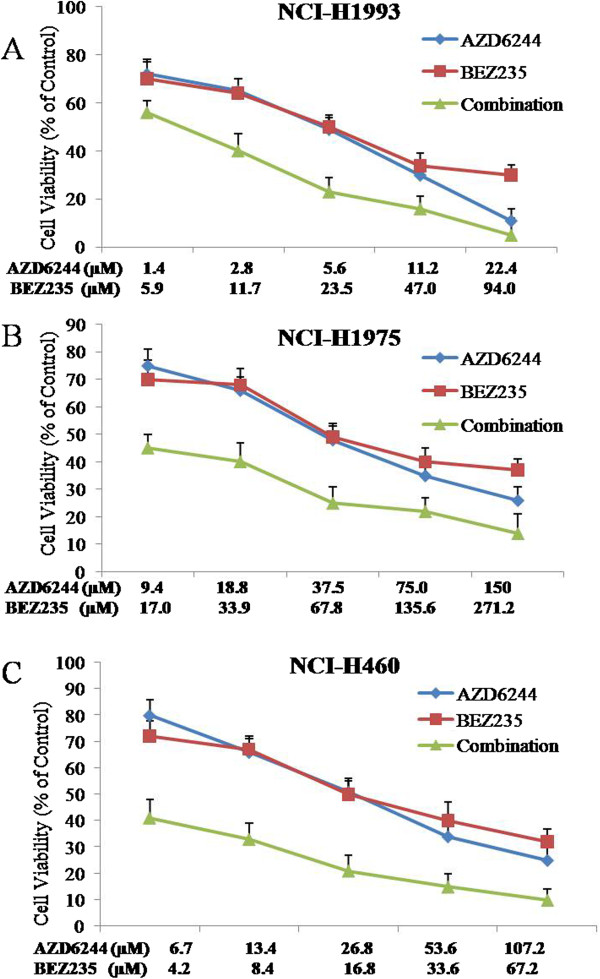
**Synergistic effects of AZD6244-BEZ235 combination therapy on cell viability.** NCI-H1993 **(A)**, NCI-H1975 **(B)** and NCI-H460 **(C)** cells were treated with AZD6244 alone, BEZ235 alone or AZD6244-BEZ235 in combination for 72 h. Results were analyzed according to the Chou-Talalay method [19]. The combination index (CI) values were calculated by using CalcuSyn software. Mean ± SD, n = 5.

The anti-proliferative effect of combining a MEK and PI3K/mTOR inhibitor was measured in NCI-H1993, NCI-H1975 and NCI-H460 cells by calculating the combination index (CI) according to the Chou-Talalay method [21] using a fixed dose ratio. Both AZD6244 and BEZ235 were introduced to cell cultures at 0.25×, 0.5×, 1×, 2× and 4× their respective IC50s in NCI-H1993, NCI-H1975 and NCI-H460 cell lines for 72 h. Cell growth in all cell lines was markedly decreased following combination treatment at multiple paired concentrations when compared with either single agent alone. The cells viability data were processed to get the CI under the corresponding effective dose (ED) in NCI-H1993, NCI-H1975 and NCI-H460 cell lines (Figure 2) by CalcuSyn software. For the NCI-H1993 cell line the following CI value was obtained: 0.4101 (ED50). For NCI-H1975 and NCI-H460 cell line the CI values were 0.02052 (ED50), and 0.0440 (ED50) respectively. The CI results suggested that AZD6244 and BEZ235 worked synergistically to produce an anti-proliferative effect in NCI-H1993, NCI-H1975 and NCI-H460 cell lines (Figures 2A-C).

### Tumor growth inhibition effect of MEK and PI3K/mTOR inhibitors in gefitinib-resistant NSCLC tumor models

**Figure 3:**
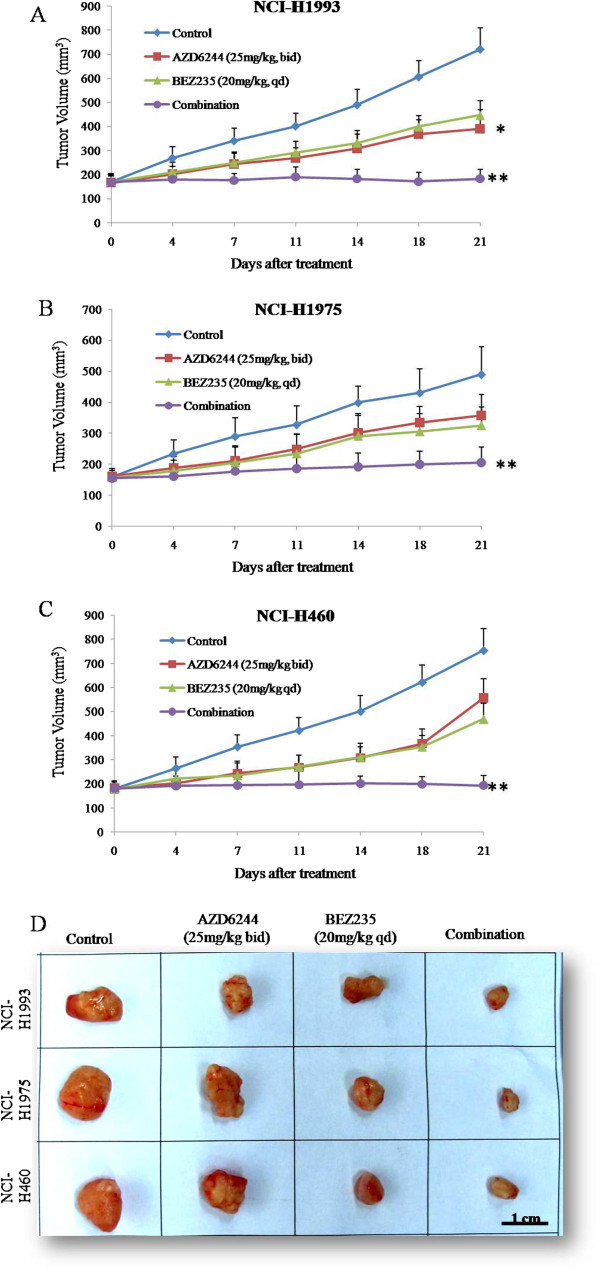
**Antitumor activity of AZD6244 and/or BEZ235 in mouse xenograft models of human tumors.** Nude mice-bearing NCI-H1993 **(A)**, NCI-H1975 **(B)**, and NCI-H460 **(C)** tumors were administered 25 mg/kg AZD6244 twice daily and/or 20 mg/kg BEZ235 once daily up to 21 days. Tumors were resected from nude mice on day 21 **(D)**. Tumor volume was measured using calipers on the indicated days. Mean ± SD, n = 10. *, *P* < 0.05 *vs* control group. **, *P* < 0.01 *vs* control group.

**Figure 4:**
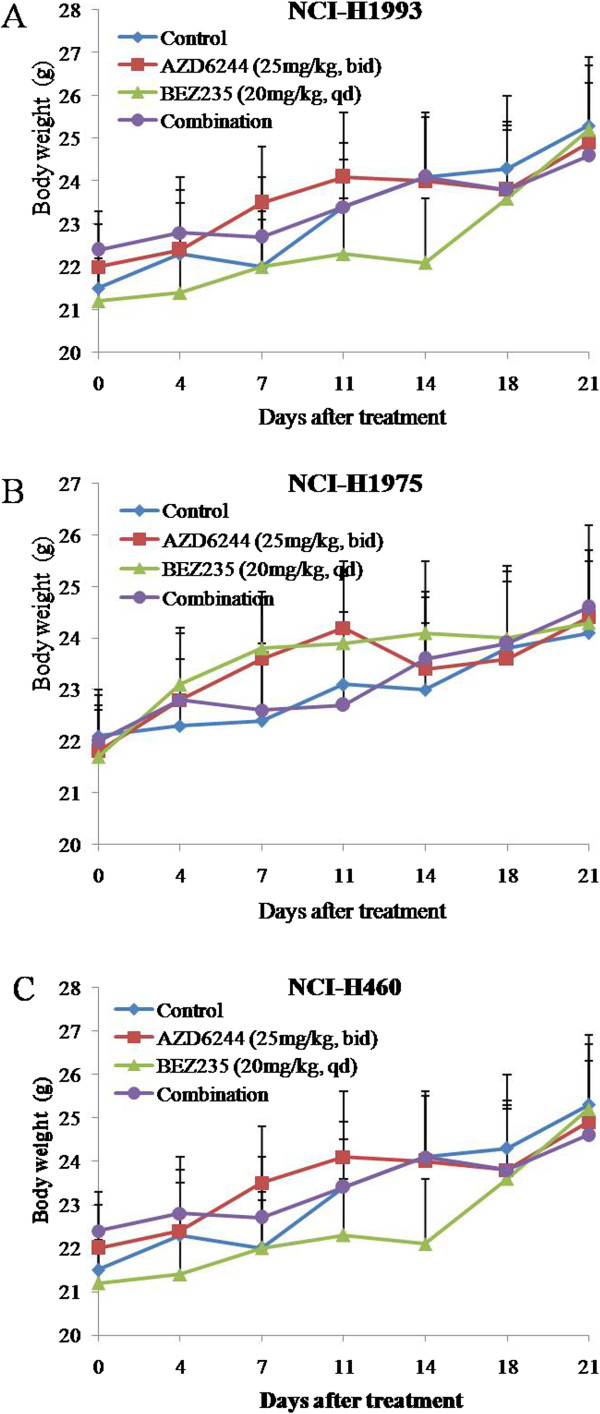
**Changes in body weight of mice treated with control group or AZD6244 and/or BEZ235.** Nude mice-bearing NCI-H1993 **(A)**, NCI-H1975 **(B)**, and NCI-H460 **(C)** tumors were administered 25 mg/kg AZD6244 twice daily and/or 20 mg/kg BEZ235 once daily up to 21 days. Body weight was measured on the indicated days. Mean ± SD, n = 10.

In order to investigate tumor growth inhibition effect of AZD6244 and/or BEZ235 *in vivo*, we used AZD6244, BEZ235, and AZD6244 plus BEZ235 to treat NCI-H1993, NCI-H1975 and NCI-H460 subcutaneous tumor models respectively for 3 weeks. As shown in Figure 3A-C, treatment with AZD6244 for 3 weeks was able to inhibit tumor growth of NCI-H1993 (T/C value 40%), but slightly inhibit tumor growth in both NCI-H1975 and NCI-H460 subcutaneous tumor models (T/C values 60% and 65%), whereas BEZ235 treatment caused an approximately 50% reduction in tumor growth in all three subcutaneous tumor models. In contrast, the combined treatments with the two drugs almost completely inhibited NCI-H1993, NCI-H1975 and NCI-H460 tumor growth at the end of the 3 weeks of therapy (Figure 3A-D). Single agent and combination treatment protocols were well tolerated by mice, with no weight loss or other signs of acute or delayed toxicity (Figure 4A-C).

### Effect of MEK and PIK3/mTOR inhibitors on signaling transduction pathways in gefitinib-resistant NSCLC tumor models

**Figure 5:**
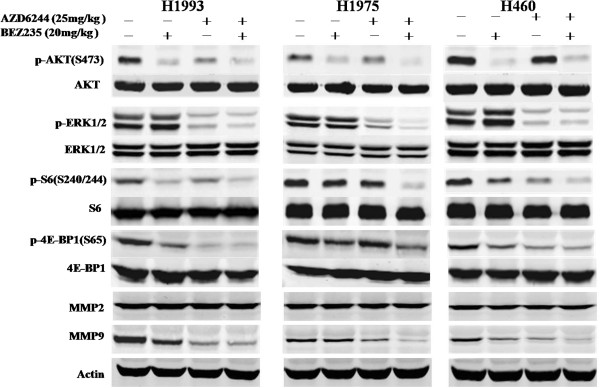
**Effects of AZD6244-BEZ235 combination therapy on PI3K/AKT and MEK/ERK pathways.** All three gefitinib-resistant tumor xenograft models were treated with the AZD6244 and/or BEZ235 for 2 h on Day 21 of the efficacy study, tumor tissues were then harvested to detect p-AKT (S473)/AKT, p-ERK (T202/Y204)/ERK, p-S6 (S240/244)/S6 and p-4E-BP1 (S65)/4E-BP1.

To assess the impact of both compounds on downstream molecules of the MEK and PI3K pathways, we used Western blot analysis to observe phosphorylation status and total protein expression in tumor tissues. The results showed that p-MEK1/2, p-ERK1/2, p-AKT, p-S6 and p-4E-BP1 appeared to be inhibited by AZD6244 and BEZ235 combination treatment, whereas the total protein levels of MEK1/2, ERK1/2, AKT, S6 and 4E-BP1 remained unchanged in each tumor model (Figure 5). Western blot analysis of downstream signals also showed treatment with AZD6244 or BEZ235 inhibited the phosphorylation of ERK1/2 or AKT in all three tumor models respectively. In addition, combined treatment with AZD6244 and BEZ235 showed greater inhibition of p-ERK1/2 and p-AKT than observed in control group or mice treated with each compound alone *in vivo* (Figure 5). Interestingly, the impact from both inhibitors on p-S6 and p-4E-BP1 levels was, alternatively, tumor model specific. For example, AZD6244 and BEZ235 alone and in combination markedly inhibited p-S6 and p-4E-BP1 expression levels in NCI-H1993 tumor models, compared with the minimal suppression observed in NCI-H460 tumor model (Figure 5). Neither AZD6244 nor BEZ235 alone suppressed p-S6 and p-4E-BP1 in NCI-H1975 tumor model. We also found that the expression of MMP-9 was significantly inhibited by AZD6244 and BEZ235 combination treatment, whereas the expression of MMP-2 was not affected by the treatment.

### Effect of MEK and PIK3/mTOR inhibitors on the expressions of Ki-67 and CD31 in gefitinib-resistant NSCLC tumor models

**Figure 6:**
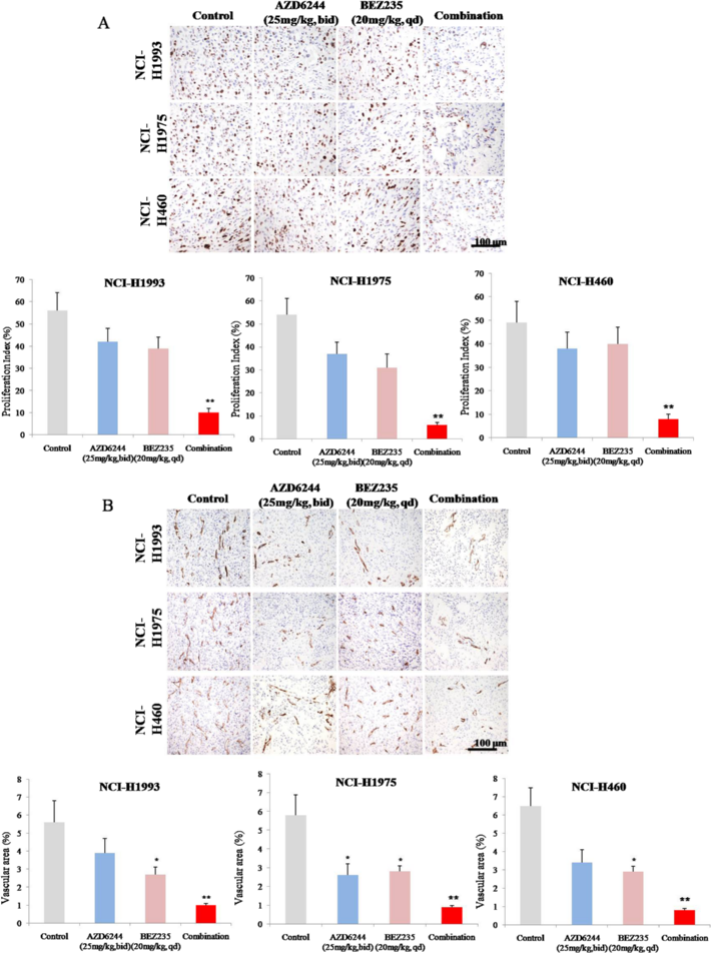
**Effects of AZD6244 and/or BEZ235 on the expressions of Ki-67 (A) and CD31 (B) in NCI-1993, NCI-1975 and NCI-H460 xenograft models.** All three gefitinib-resistant tumor xenograft models were treated with the AZD6244 and/or BEZ235 for 2 h on Day 21 of the efficacy study, tumor tissues in each group were resected and immunostained with anti-Ki67 and anti-CD31 antibodies. N = 10, *, *P* < 0.05 vs vehicle group. **, *P* < 0.01 *vs* vehicle group.

To characterize the mechanism of tumor growth inhibition observed in our gefitinib-resistant NSCLC tumor models by AZD6244 and BEZ235, lung tumor tissues were assessed by evaluating Ki-67 expression using immunohistochemical analyses. We observed active cell proliferation in NCI-H1993 tumor model, with a 56% proliferation index (Figure 6A). Monotherapy with AZD6244 or BEZ235 slightly decreased the percentage of Ki-67-positive proliferating tumor tissues, with proliferation indices of 42% and 39%, respectively (Figure 6A). Combined treatment with AZD6244 and BEZ235 markedly decreased the percentage of Ki-67-positive proliferating tumor tissues to 10%, consistent with the marked inhibition of ERK1/2 and AKT phosphorylation. We also found the similar results in NCI-H1975 and NCI-H460 tumor models (Figure 6A).To evaluate the potential antiangiogenic mechanism of AZD6244 and BEZ235, gefitinib-resistant NSCLC tumor tissues were analyzed by immunostaining for CD31 (platelet endothelial cell adhesion molecule 1). The results showed BEZ235 significantly decreased the vascular density of all three gefitinib-resistant NSCLC tumors, whereas AZD6244 monotherapy had only a mildly effect upon lung tumor angiogenesis. The antiangiogenic effects AZD6244 and BEZ235 were markedly increased when they were combined (Figure 6B).

### MEK and PIK3/mTOR inhibitors had no effect on caspase-3, 8 and 9 activities in gefitinib-resistant NSCLC tumor models

**Figure 7:**
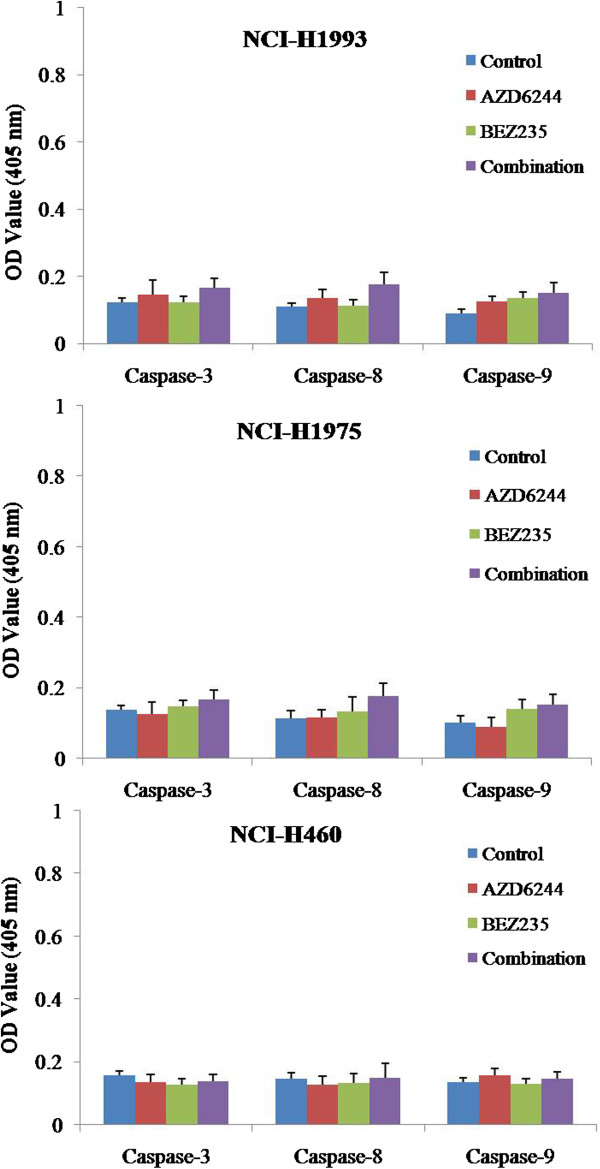
**Effects of AZD6244 and/or BEZ235 on the activities of caspase-3, 8 and 9 in NCI-1993, NCI-1975 and NCI-H460 xenograft models.** All three gefitinib-resistant tumor xenograft models were treated with the AZD6244 and/or BEZ235 for 2 h on Day 21 of the efficacy study, tumor tissues in each group were resected and measured by caspase colorimetric protease kits. N = 10.

In order to investigate whether AZD6244 and/or BEZ235 would induce apoptosis in gefitinib-resistant NSCLC tumor models, activity of caspase-3, 8 and 9 were measured by the colorimetric assay. The results showed that AZD6244 and/or BEZ235 had no effect on caspase-3, 8 and 9 activities in all three gefitinib-resistant NSCLC tumor models (Figure 7).

## Discussion

Although advances have been made in cancer treatment with the development of selective molecular targeted therapies, several relevant issues for their optimal and effective use remain unsolved. Recent studies have demonstrated that the EGFR-TKI gefitinib and erlotinib are associated with a high response rate and prolong progression-free survival in patients with EGFR mutant lung cancer [22]. Responders to these agents, however, later relapse after acquiring EGFR-TKI resistance, making it urgent to develop novel therapeutic agents that can overcome acquired resistance to EGFR-TKI. Current clinical approaches to combat resistance in lung adenocarcinoma include irreversible and mutant-selective inhibitors of EGFR, combination of cetuximab and afatinib [23] and combination of an EGFR inhibitor with a drug targeting a resistance pathway, such as the combination of gefitinib and a MET inhibitor [24]. However, alternative RTK pathways that are activated following EGFR inhibition are another area for investigation. These alternative pathways may bypass or evade inhibition of EGFR signaling, thereby enabling combinations of agents to simultaneously attack multiple molecular targets for cancer growth inhibition. One potential solution to overcome multiple mechanisms of resistance is to target downstream pathways. In this study, we show that the combination of a selective MEK inhibitor and a PI3K/mTOR inhibitor is effective against tumor cell lines refractory to gefitinib by three different mechanisms: an *EGFR* gatekeeper T790M mutation, *MET* amplification, and *KRAS/PIK3CA* mutation. To our knowledge, this is the first report of the effects of MEK TKI with PI3K/mTOR TKI therapy in gefitinib-resistant models of NSCLC.

MEK is a potentially relevant molecular therapeutic target since it is the activated downstream of the axis RAS/RAF proteins and in turn activates ERK to induce cell proliferation. Therefore, several selective MEK inhibitors have been developed [25] and more than ten MEK inhibitors have entered early clinical trial evaluation [26]. Unfortunately, clinical activity as single agent has been rarely observed with MEK inhibitors in gefitinib-resistant NSCLC patients [27]. The benefit of blocking an individual pathway has been largely limited by the presence of a compensatory feedback loop between PI3K and MEK. For example, inhibition of the MEK pathway results in activation of the PI3K pathway [28], and PI3K activation mediates resistance to MEK inhibition [29]. In our study, in order to circumvent this compensatory feedback, concurrent blockade of the two pathways has been tested, and synergy in antitumor effects was detected, providing the rationale for phase I clinical trials. Moreover, early signs of clinical benefit have been reported in advanced cancer by a retrospective analysis on patients receiving agents that target both pathways [30].

Gatekeeper mutations, the T790M mutation in *EGFR* associated with resistance to gefitinib [31], are common mechanisms by which tumor cells acquire resistance to molecularly targeted drugs. Although irreversible EGFR-TKIs, including BIBW2992, have been developed to overcome T790M-mediated resistance to gefitinib [32], recent clinical trials have failed to show that monotherapy with irreversible EGFR-TKIs has benefits in patients with NSCLC refractory to gefitinib [33]. This may be due, at least in part, to the low selectivity of this class of compounds to wild-type and mutant *EGFR*. In addition, because HGF overexpression was frequently observed in tumors with the gatekeeper T790M mutation [34], monotherapy with a mutant-selective EGFR-TKI may not be sufficient to inhibit the growth of tumors with acquired resistance to gefitinib. Our findings suggest that the combination of a MEK-TKI and a PIK3/mTOR-TKI may be effective in controlling these resistant tumors.

Many *KRAS*-mutant cancer cells have been shown to be sensitive to MEK inhibitors, [35] and *KRAS* mutations can be detected in up to 30% of lung cancers, dependent upon histology and ethnicity [36-38], suggesting that a subset of lung cancers would likely be highly sensitive to AZD6244. Our finding that AZD6244 was effective in one distinct *KRAS* mutant human lung cancer NCI-H460 models supports and validates this hypothesis. Although monotherapy with AZD6244 resulted in antitumor and some antiangiogenic effects in all of our lung cancer models, the antitumor effects were more apparent in the NCI-H1993 lung adenocarcinoma model. The increased antitumor efficacy observed in this model is associated with differences in the inhibitory effect of p-AKT signaling pathway in NCI-H1975 and NCI-H460 lung tumors. However, additional studies are needed to elucidate this phenomenon.

In this study, we evaluated therapy directed against MEK and PI3K/mTOR in distinct gefitinib-resistant NSCLC xenograft models. MEK or PI3K/mTOR inhibition resulted in antitumor effects for these gefitinib-resistance NSCLC models by blocking key intracellular pathways controlling cell proliferation and survival as demonstrated both *in vitro* and *in vivo.* Surprisingly, PI3K/mTOR inhibition by BEZ235 also suppressed lung tumor angiogenesis and targeted both MEK and PI3K/mTOR activation in lung tumors, resulting in substantial antiangiogenic effects, which may due to the significantly reduced expression of MMP-9 in tumors. We concluded that the combination of a selective MEK inhibitor and a PI3K/mTOR inhibitor was effective in suppressing the growth of gefitinib-resistant tumors caused by *EGFR* T790M mutation, *MET* amplification, and *KRAS*/*PIK3CA* mutation. These findings represent a promising strategy for the treatment of gefitinib-resistant NSCLC and provide a strong basis for the design of clinical trials for this purpose.

## Competing interests

The authors declare that they have no competing interests.

## Authors’ contributions

YQQ conceived and designed the experiments. XXW, YHY, YY and HL performed the experiments. XXW, YHY and DDM analyzed the data. XXW, YHY and HL wrote the paper. YQQ supervised the whole experimental work and revised the manuscript. All authors read and approved the manuscript.
